# Prediction of Depression in Individuals at High Familial Risk of Mood Disorders Using Functional Magnetic Resonance Imaging

**DOI:** 10.1371/journal.pone.0057357

**Published:** 2013-03-06

**Authors:** Heather C. Whalley, Jessika E. Sussmann, Liana Romaniuk, Tiffany Stewart, Martina Papmeyer, Emma Sprooten, Suzanna Hackett, Jeremy Hall, Stephen M. Lawrie, Andrew M. McIntosh

**Affiliations:** 1 Division of Psychiatry, University of Edinburgh, Edinburgh, United Kingdom; Beijing Normal University, China

## Abstract

**Objective:**

Bipolar disorder is a highly heritable condition. First-degree relatives of affected individuals have a more than a ten-fold increased risk of developing bipolar disorder (BD), and a three-fold risk of developing major depressive disorder (MDD) than the general population. It is unclear however whether differences in brain activation reported in BD and MDD are present before the onset of illness.

**Methods:**

We studied 98 young unaffected individuals at high familial risk of BD and 58 healthy controls using functional Magnetic Resonance Imaging (fMRI) scans and a task involving executive and language processing. Twenty of the high-risk subjects subsequently developed MDD after the baseline fMRI scan.

**Results:**

At baseline the high-risk subjects who later developed MDD demonstrated relatively increased activation in the insula cortex, compared to controls and high risk subjects who remained well. In the healthy controls and high-risk group who remained well, this region demonstrated reduced engagement with increasing task difficulty. The high risk subjects who subsequently developed MDD did not demonstrate this normal disengagement. Activation in this region correlated positively with measures of cyclothymia and neuroticism at baseline, but not with measures of depression.

**Conclusions:**

These results suggest that increased activation of the insula can differentiate individuals at high-risk of bipolar disorder who later develop MDD from healthy controls and those at familial risk who remain well. These findings offer the potential of future risk stratification in individuals at risk of mood disorder for familial reasons.

## Introduction

Mood disorders, comprising bipolar disorder (BD) and major depressive disorder (MDD), are among the top ten causes of disability worldwide [1], [2]. They are known to be heritable, with overlapping genetic architecture [3]–[6]. First degree relatives of affected bipolar patients are at more than a ten-fold higher risk of developing BD than members of the general population, and more than three-fold increased risk of developing MDD [7]. Neuroimaging studies of mood disorders have identified dysfunction in a wide network of regions, including prefrontal, limbic and paralimbic regions, including the insula cortex [2], [8], [9]. Typically, these are explored using emotional processing paradigms, where over-activation is commonly reported to emotional stimuli [10]. More recently however there are also increasing number of studies reporting dysfunctional activation of mood processing regions in response to cognitive tasks [11], [12]. It is however unclear whether these abnormalities are evident prior to onset, and whether they predict those who later develop a mood disorder. To answer these issues it is necessary to conduct prospective longitudinal studies of young unaffected relatives.

To realise this aim, we examined a cohort of unaffected young individuals at high familial risk of BD. These individuals were at high risk because they had first and/or second degree relatives with BD. As BD and MDD share genetic liability, this cohort is at high-risk of both unipolar and bipolar mood disorders. At baseline, participants were scanned using a cognitive sentence completion task probing executive and verbal fluency processes which are known to be disrupted in mood disorder [12]. We previously reported that the high risk group demonstrated increased activation of the amygdala in the context of this cognitive task versus healthy controls [12]. The task has been shown to differentiate patients with BD, and those at increased familial risk of BD, from healthy controls [9], [12], and to distinguish those at risk of a schizophrenia with and without depressive features [13]. All individuals were assessed longitudinally and categorised according to their clinical status at follow-up 2 years later. We hypothesised that there would be activation differences in those who subsequently developed a mood disorder in regions commonly associated with these conditions and mood regulation. Since there is a lack of prior studies examining neuroimaging measures in individuals converting to mood disorder, we based our hypotheses on studies examining patient samples versus controls. Since the regions commonly reported in patient groups include large distributed networks, including prefrontal, limbic and paralimbic regions, we conservatively report results corrected for multiple comparisons at the whole brain level.

## Methods

### Study population

The study was approved by the Multi-Centre Ethics Committee for Scotland, Committee A. Participants were recruited as part of the Scottish Bipolar Family Study [12]. Individuals with a diagnosis of bipolar I disorder were identified by psychiatrists across Scotland. Each affected subject was asked to identify members of close family aged 16–25 years. The diagnosis of affected subjects was confirmed with the OPCRIT [14] symptom checklist using data from clinical notes and the structured clinical interview for DSMIV (SCID). Following informed consent, unaffected individuals with at least one first degree, or two second degree relatives with bipolar I disorder were invited to participate. The majority of high-risk individuals had a first degree relative with the exception of three high-risk individuals from the well group who had affected second degree relatives only. Unaffected, unrelated comparison subjects with no personal or family history of bipolar disorder were identified from the social networks of the high-risk subjects and matched for age, sex and premorbid IQ to the high-risk group. Comparison subjects were also screened using the SCID. Exclusion criteria for both groups included a personal history of major depression, mania or hypomania, psychosis, or any major neurological or psychiatric disorder, a history of substance dependence, learning disability, or any history of head injury that included loss of consciousness and any contraindications to MRI. After complete description of the study to the subjects, written informed consent was obtained for all participants. Participants who declined to participate were not disadvantaged in any way by not participating in the study.

### Clinical assessments

Baseline clinical assessments were conducted at the time of the first functional scan. Follow-up assessments were conducted on individuals who returned for a second assessment approximately 2 years later. For subjects who did not return for a second assessment diagnostic status was determined through written contact with the National Health Service (NHS) (n = 14 controls and n = 19 bipolar high risk subjects who consented to this data being obtained). Clinical interviews at both assessments were conducted by two trained psychiatrists (AMM, JES). Participants were re-interviewed at follow-up using the SCID to determine whether they had developed a diagnosis of mood disorder; namely MDD or BD. On the basis of their follow-up assessment, or on information provided by the case notes, the bipolar high-risk group was split into those who remained well, and those who subsequently developed MDD or BD. The mean interval in months between assessments was 24.79 (SD 2.64), 25.47 (SD 4.15), 26.31 (SD 3.57) for controls, high-risk well, and high-risk who subsequently became ill respectively, p = 0.35. At baseline, current manic and depressive symptoms were rated using the Young Mania Rating Scale [15] and Hamilton Depression Rating Scale (HAM-D) [16], and estimates of trait liability to mood disorder were measured using the TEMPS-A cyclothymia scale [17], and neuroticism and extraversion were measured using the NEO-FFI [18]. Statistical analysis of demographic data was conducted using one-way ANOVAs or chi-squared where appropriate in SPSS version 19. For the clinical assessments and measures of temperament, comparison of groups was conducted using Kruskal-Wallis tests.

### Experimental paradigm

Participants performed the verbal initiation section of the Hayling sentence completion test [19] in the scanner [12], [20]. Subjects were shown sentences with the last word missing and asked to silently think of an appropriate word to complete the sentence and press a button when they had done so, generating a within-scanner measure of reaction time. Sentences were selected from a set of completion norms [21]. The task had four levels of difficulty according to the sentence context. Sentences were presented in blocks of fixed difficulty, each block lasted 40 seconds and included eight sentences. Block order was pseudo-random and each block was repeated four times using different sentences. This design allowed a standard subtraction (sentence completion versus baseline) and parametric analysis (examining areas of increasing activation with increasing task difficulty). Scanning procedure, image processing and analysis details are provided in supplementary material (Text S1).

Immediately after scanning, subjects were given the same sequence of sentences on paper and requested to complete each sentence with the word they first thought of in the scanner. ‘Word appropriateness’ scores were determined from the list of sentence completion norms [21] which provides respective probabilities of possible responses. Mean scores for word appropriateness and reaction time were determined.

### Main analysis

For each contrast of interest (sentence completion versus baseline and the parametric contrast), one contrast image per individual was entered into a second level random-effects analyses. Analysis was conducted on individuals with known clinical status (either from the second assessment or through information from the NHS) using a factorial design, with group as the single factor (three levels: controls, high-risk remaining well and high-risk subsequently ill). From the available baseline scan data (110 bipolar high risk and 70 controls [12], the status of 10 bipolar high risk and 8 controls was not able to be established and these individuals were excluded. Controls who developed a mood disorder were also excluded (n = 4, all MDD). The majority of the ill group (n = 20) developed MDD, however one individual developed BD type I and one individual developed BD type II. Since only 2 individuals had developed a mood disorder with a manic component, the main analysis focussed on individuals who had developed MDD only. Subsequent analysis including these 2 individuals is also included for completeness.

Statistical maps were thresholded at the standard level of p = 0.001 uncorrected, and regions were considered significant at p<0.05 cluster level corrected for multiple comparisons across the whole brain. All p values are at the cluster level corrected for multiple comparisons. Results are presented as (p value, K_E_ indicating the number of voxels within a cluster, and co-ordinates in x, y and z dimensions). Co-ordinates are reported in MNI (Montreal Neurological Institute) convention. All images are overlaid onto standard brain in MNI space using Mango software package (http://ric.uthscsa.edu/mango). Standard Receiver Operating Characteristic curves were generated for clusters of interest using the diagnostic outcome of MDD using ‘R’ software.

### Relationship to trait liability measures

We also examined associations between the activation differences between the groups and measures of trait liability to mood disorder, namely cyclothymia scores, neuroticism and extraversion. This was performed using correlation analysis on the extracted clusters in SPSS. In each case, we predicted that any activation differences would be related to trait liability to BD (as measured by increasing cyclothmia scores) or MDD (as measured by increasing neuroticism or decreasing extraversion).

### Analysis of potential confounders

To address the potential role of symptoms at the time of the scan, relationships between activation and measures of depression and mania from the HAM-D and YMRS were examined. We also examined relationships between insula activation and measures of weekly alcohol consumption and illicit substances. As above these were performed using data from the extracted clusters in SPSS. Finally, we performed an additional analysis including only one family member per group chosen at random in order to exclude factors related to the effects of multiple family members.

## Results

### Demographic, clinical, and behavioural measures

Of the 98 high-risk individuals with baseline imaging and genetic data, 20 subsequently developed MDD. The groups are referred to as HR well (n = 78), HR who developed MDD (n = 20) and HC (healthy controls, n = 58). Demographic details are presented in Table 1. There were no significant differences between the groups in terms of age, gender, handedness, substance misuse or IQ, nor for any of the task-related performance measures of within-scanner reaction time or word appropriateness.

**Table 1:** Demographics, clinical, behavioural and temperament measures.

	Controls (n = 58)		High-risk well (n = 78)		High-risk who developed MDD (n = 20)		Significance
	Mean/median	St dev/IQR	Mean/median	St dev/IQR	Mean/median	St dev/IQR	P value (F/χ^2^)
**Demographics**							
Mean age (yrs)	20.78	(2.39)	21.12	(3.67)	20.59	(2.94)	0.74 (0.30)
Gender (M∶F)	25∶33	-	42∶36	-	8∶12	-	0.34 (2.14)
Handedness (R∶Other)	55∶3	-	68∶10	-	20∶0	-	0.22 (5.74)
Mean NART IQ	109.00	(7.45)	107.76	(15.49)	107.35	(6.88)	0.80 (0.23)
**Clinical measures** *							
YMRS	0	(0)	0	(0)	0	(0.75)	0.13 (4.04))
HAM-D	0	(0)	0	(2)	1.50	(5.75)	<0.01 (11.15)
**Behavioural measures**							
Reaction time (ms)	2474	(603)	2540	(697)	2558	(569)	0.54 (0.69)
Mean word appropriateness score	3.02	(0.52)	2.91	(0.65)	2.98	(0.47)	0.81 (0.21)
**Temperament and personality measures** * **: (TEMPS-A)**							
Cyclothymia	1.00	(3.00)	2.00	(2.50)	6.00	(7.25)	<0.01 (13.91)
Depressive	0.00	(2.00)	0.00	(1.00)	2.50	(4.00)	<0.01 (9.45)
Irritability	1.00	(2.00)	1.00	(2.00)	2.00	(2.75)	0.01 (8.88)
Hyperthymia	3.00	(3.00)	2.00	(2.00)	2.00	(3.50)	0.25 (2.78)
Anxious	1.00	(2.00)	0.00	(1.00)	1.50	(3.00)	0.07 (5.27)
Total score	7.00	(9.50)	6.00	(6.00)	14.50	(13.50)	<0.01 (14.21)
**NEO – Five Factor Inventory:**							
Neuroticism	20.45	(8.74)	21.18	(9.47)	32.06	(10.35)	<0.01 (10.36)
Extraversion	31.13	(6.17)	29.22	(6.31)	24.88	(7.54)	0.03 (5.96)
Openness	28.87	(6.05)	27.14	(6.42)	29.31	(3.91)	0.19 (1.67)
Agreeableness	32.34	(5.21)	32.11	(6.52)	29.19	(7.13)	0.18 (1.74)
Conscientious-ness	29.19	(6.26)	27.81	(7.64)	23.94	(8.23)	0.04 (3.25)
**Lifetime substance misuse N (%)**							
Alcohol (U/week)	15.47	16.79	14.27	13.88	10.60	11.69	0.50 (0.81)
Tobacco	13	22.4	22	28.2	8	40	0.34 (2.19)
Cannabis	34	58.6	51	65.4	13	65	0.78 (0.49)
Stimulants	12	20.7	20	25.6	8	40	0.25 (2.75)
Hallucinogens	6	10.3	12	15.4	3	15	0.71 (0.70)
Opiates	1	1.7	3	3.8	0	0	0.22 (3.00)
Sedatives	2	3.4	7	9.0	1	5.0	0.43 (1.70)

* Kruskal-Wallis tests, median and interquartile range presented for skewed variables.

There were however significant differences between the groups for baseline clinical measures of depression from the HAM-D (p = 0.004). There were also significant differences at baseline between the groups for measures of cyclothymia (p = 0.001). For both these measures the HR who developed MDD scored the highest. These were statistically significant between the HC and HR who developed MDD, as well as between the HR well and HR who developed MDD. The only measure that was significantly different between the HC and HR well was the measure of depression from the HAM-D (p = 0.04), with higher scores in the HR well group. There were also significant differences between the groups for personality-based measures of trait liability to depression; for neuroticism (p<0.001) and extraversion (p = 0.003). Pair-wise comparisons indicated significant differences between HC and HR who developed MDD, and between HC and HR well. Further details of the pair-wise comparisons can be found in Table S1.

### Task-related brain activation patterns

All subjects demonstrated the expected patterns of brain activation and behavioural responses indicating subjects were performing the task appropriately in the scanner [12], [20], [22], and see Figure S1. Regions activated across the groups for the sentence completion versus baseline contrast included the left medial and lateral prefrontal regions, left lateral temporal cortex, sub-cortical structures, left lateral parietal cortex, occipital lobes bilaterally, and right cerebellum. Regions of deactivation included bilateral insula cortex and midline fronto-parietal regions. For the parametric contrast, areas of activation including left lateral and medial prefrontal cortex, left lateral temporal cortex, and right cerebellum, see Figure S2.

### Between group differences in activation

For sentence completion versus baseline, there were no significant differences between the three groups.

**Figure 1:**
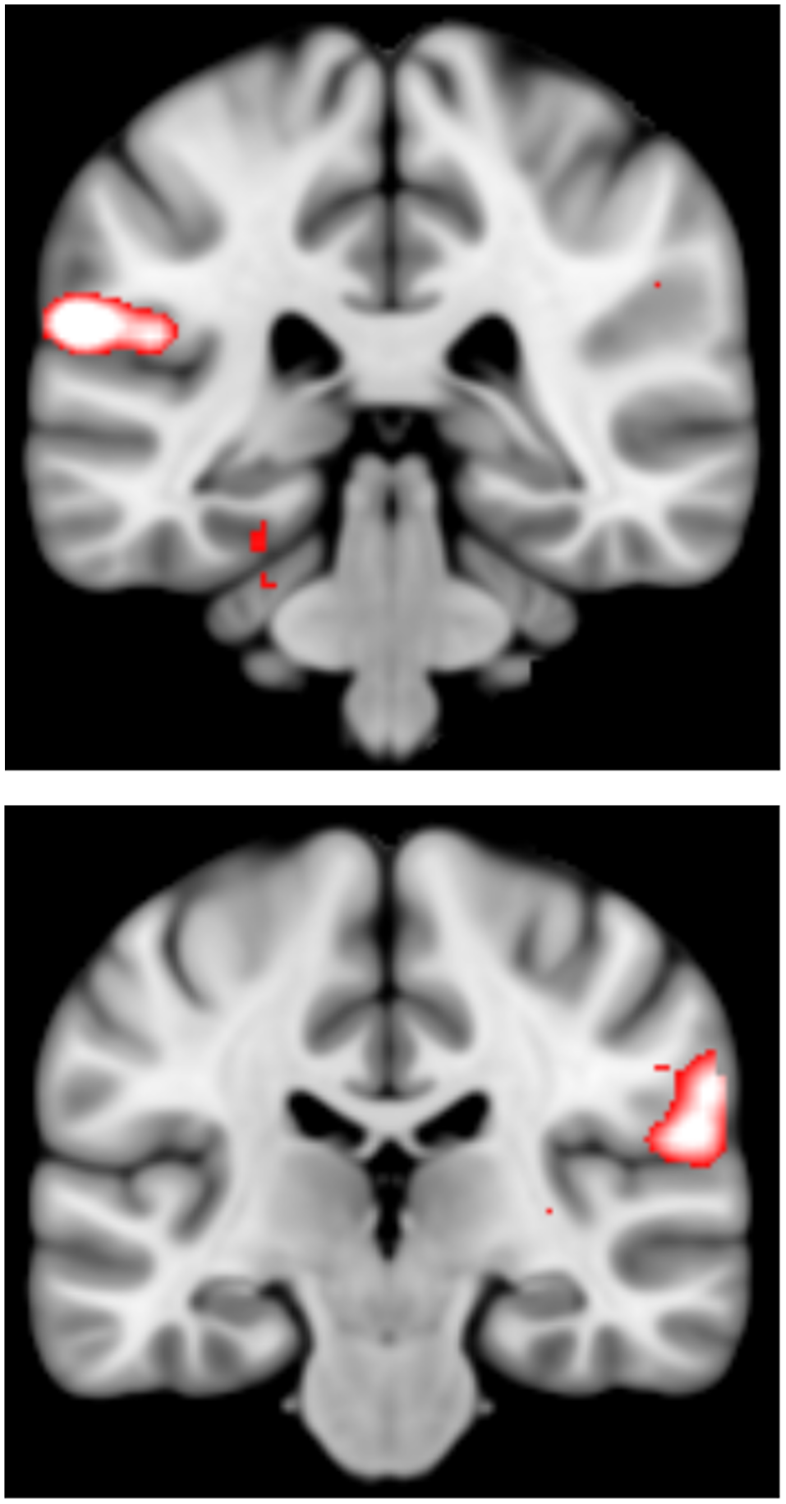
Group difference in bilateral insula cortex. Figure 1 depicts differences between the healthy controls and HR who developed MDD in the (a) left and (b) right insula cortex. Images are overlaid onto standard brain in MNI space using Mango software package (http://ric.uthscsa.edu/mango). Map represents T-statistic images thresholded equivalent to p = 0.001, see methods for further details (scale T = 3 to 5).

**Figure 2:**
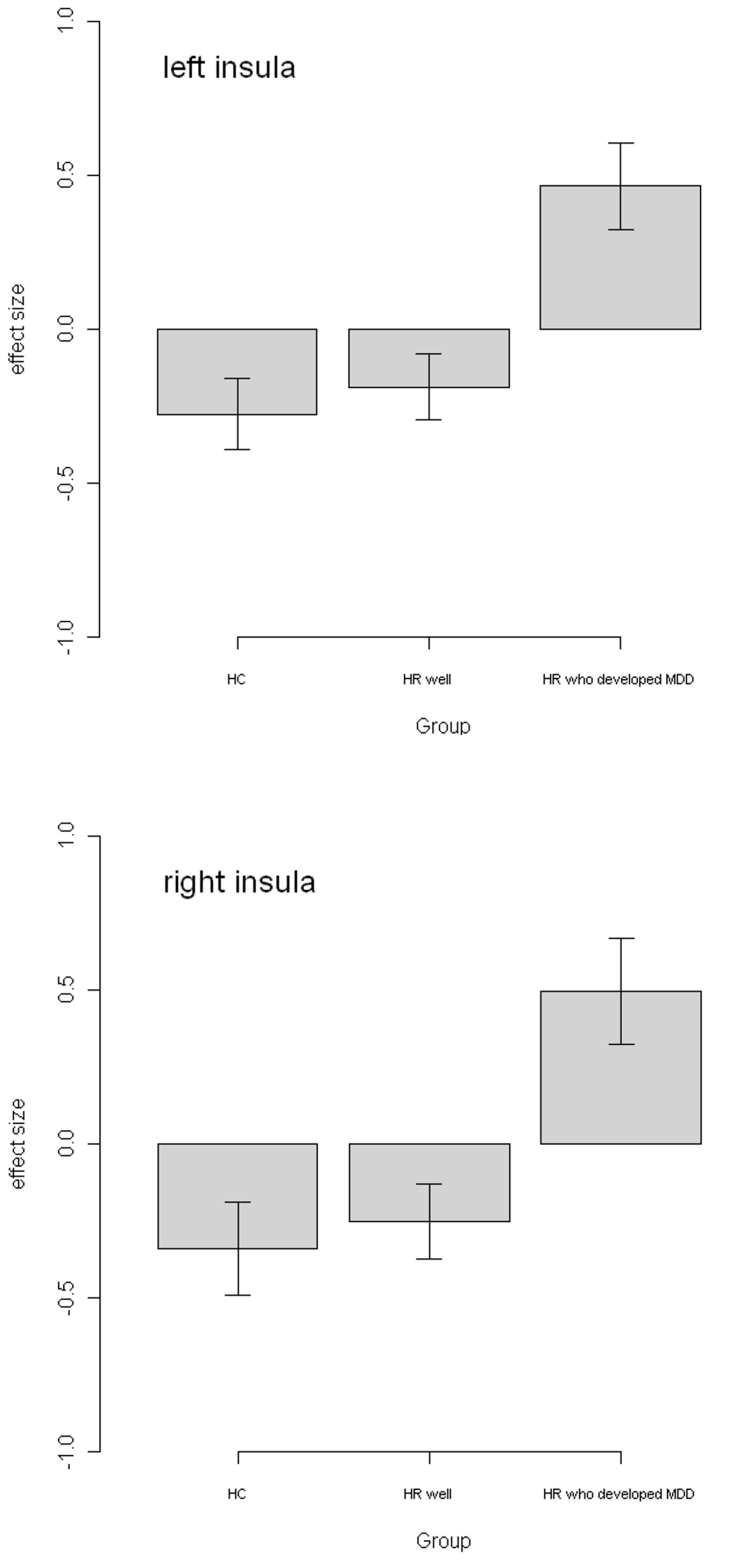
Graph of extracted data for insula clusters. Figure 2 depicts graphs of extracted data for the two cluster of significant difference between the groups in (a) left and (b) right insula cortex.

**Figure 3:**
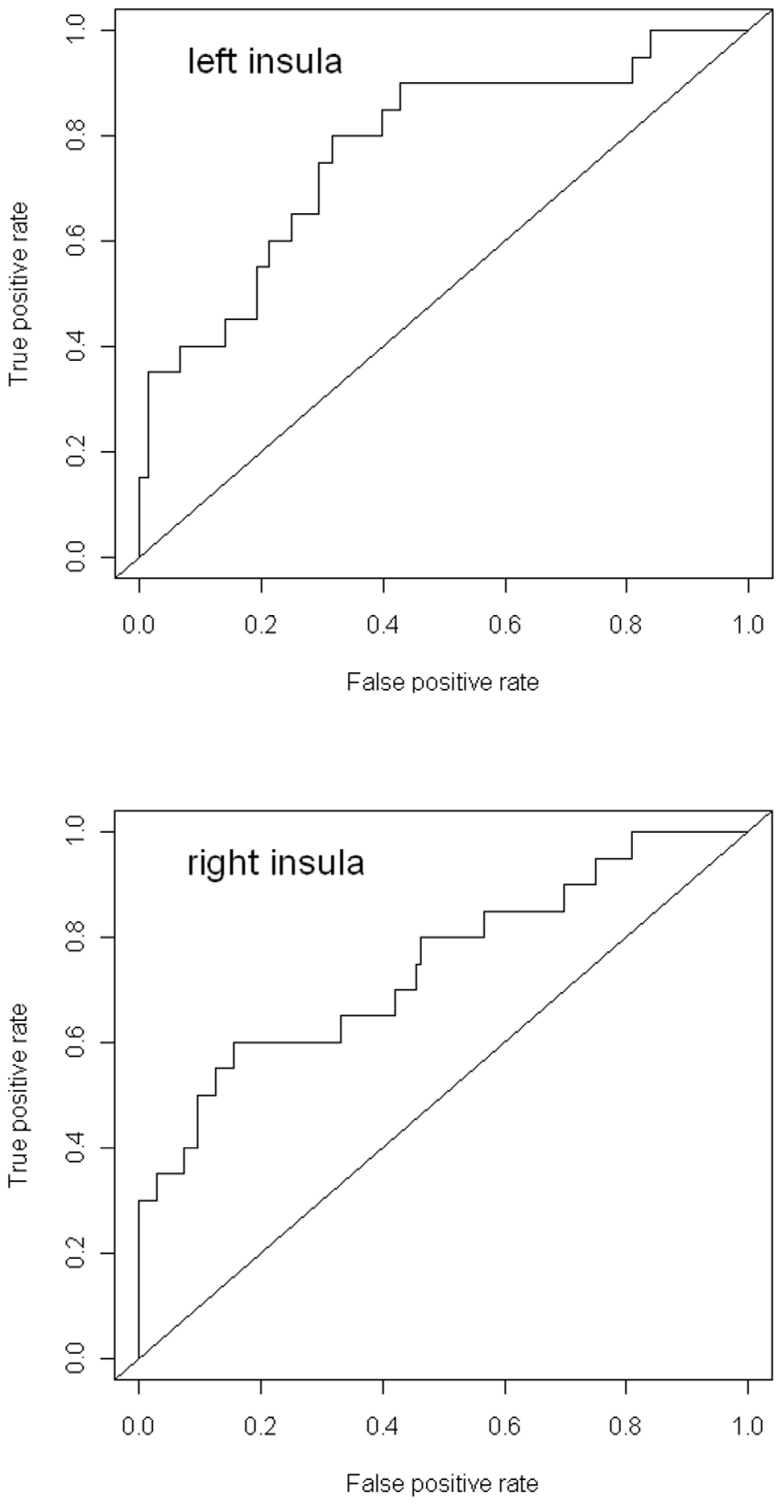
Standard Receiver Operating Characteristic plots. Plots are displayed for (a) left and (b) right insula using the diagnostic outcome of MDD. These reflect increasing activation with increasing task difficulty. Area under the curve was 0.77 and 0.73 for left and right respectively, see text for further details.

There were however significant differences between the groups for the linear ‘parametric’ contrast of brain activation with increasing task difficulty. These occurred between the HC versus HR who developed MDD in the bilateral insula, extending laterally to inferior parietal regions (p = 0.011, K_E_ = 288, Z = 4.79, x = −60, y = −36, z = 22 and p = 0.017, K_E_ = 262, Z = 4.06, x = 60, y = −24, z = 20, for left and right respectively, p values are cluster corrected at the whole brain level), and between the HR well versus HR who developed MDD in similar regions (p = 0.021, K_E_ = 247, Z = 4.16, x = −46, y = −36, z = 22 and p = 0.021, K_E_ = 247, Z = 4.16, x = 64, y = −30, z = 16, p values cluster corrected at the whole brain level). These findings are displayed in Figure 1a, and b. Graphs of extracted data are displayed in Figures 2a and b, along with standard ROC curves (Figure 3a and b) where the area under the curve was 0.77 and 0.73 for the left and right insula respectively.

### Relationship to trait liability measures

There were significant associations between the main clusters identified by the above analysis and baseline measures of neuroticism (p = 0.04, r = 0.16; p = 0.06, r = 0.15 for right and left respectively), extraversion (p = 0.003, r = −0.24; p = 0.008, r = −0.21 for right and left respectively), and cyclothymia (p = 0.03, r = 0.17; p = 0.03, r = 0.17 right and left respectively) across all subjects. In each case these correlations went in the direction expected, namely that increasing insula activation was also associated with increasing trait liability to both BD and MDD.

### Analysis of potential confounders

There were no significant correlations between activation in the clusters above and baseline symptoms at the time of the scan as measured using the HAM-D and YMRS, either across all subjects, or within the three groups separately. There were also no significant relationships between insula activation and average weekly alcohol consumption, or for groups split according to whether or not they had ever used any of the substances as listed in the demographics table. We performed 3 additional group comparison analyses to fully explore the above findings. (i) We repeated the group comparison including the 2 participants who developed BD in with the HR individuals who developed MDD. The left cluster remained significant (p = 0.02), and the right cluster fell just below statistical significance (p = 0.06). (ii) We also repeated the analysis excluding relatives from the familial group (i.e. only including one individual per family, selected randomly), leaving group sizes of n = 67 HR well and n = 16 HR who developed MDD. The left cluster remained significant (p = 0.03), and the right cluster fell just below significance (p = 0.08). (iii) Finally we analysed the dataset including only those subjects who had been interviewed ‘face-to-face’ by a trained psychiatrist at the follow up assessment (SCID). The results remained statistically significant for both left and right sides (p<0.05, p = 0.03 for left and right respectively).

## Discussion

We have demonstrated that increased activation of the bilateral insula cortex occurs in unaffected individuals at high familial risk of bipolar disorder who later develop MDD. This pattern of activation differentiated them from healthy controls and from other individuals at high risk who did not become unwell, and was un-confounded by illness and psychotropic medication. At baseline those who subsequently developed MDD also demonstrated significantly increased scores for depression, cyclothymia, neuroticism and extraversion. It is important to stress that at the time of the baseline scan none of the individuals met criteria for a mood disorder, and none were taking antidepressants or mood stabilisers. Activation differences in the bilateral insula cortex also correlated with personality and temperament measures of trait liability to mood disorder, but not with depressive symptoms at the time of the scan. The increased insula activation remained significant after including the two BD individuals, after removing related subjects from the analyses, and after restricting the analysis to only those subjects with two SCID interviews with a psychiatrist. These findings suggest that there is a pattern of abnormal brain activation that predicts MDD in young individuals at high familial risk which is related to trait liability measures of mood disorder but not to current symptomatic state.

The insula is part of a network of regions that plays a key role in the regulation of emotion, including emotional processing [8], response inhibition [23], and in the subjective experience of emotion [24]. Both structural and functional imaging studies have implicated the insula in depression [25], [26], including volumetric reductions, and correlations between the severity of depressive symptoms and volumetric loss [27]. Functional imaging has also indicated an association between insula activation and levels of neuroticism [28]. Neuroticism is not only a core personality trait associated with BD and depression, but is also a robust predictor of MDD [29], and therefore highly relevant to the current findings [29], [30].

In the current study we report a relative over-activity of the insula in response to increasing task difficulty in HR subjects who developed MDD 2 years later. The extracted data (Figure 2) shows that the controls subjects and the HR individuals who remained well demonstrated decreasing activation of the insula with increasing task difficulty, but that the HR individuals who subsequently developed MDD did not exhibit this normal pattern of inhibitory response. The insula is part of an extended salience network of regions involved in self-reflective processing, conscious experience and interoceptive awareness. It is thought to be involved in processing salience and recruiting either the relevant emotional brain areas or switching to central executive regions [31]. The current finding may therefore reflect a graded failure to disengage the insula with increasing cognitive demand in those who developed MDD. Indeed, ‘resting state’ studies, which isolate such regions, have reported differences in insula activation in patients with depression [25]. Further, this fits with cognitive models of depression where there is suggested to be a disproportionate allocation of resources to the internal experience of emotional responses, and a withdrawal of responses from higher order cognitive processes involved in the reappraisal of negative emotions [32].

Another region considered of prime importance in mood disorders is the amygdala. The amygdala and insula are highly interconnected structures [33]. In our previous study of the same cohort we reported significant differences at baseline in amygdala activation between healthy controls and all those at familial risk of BD [12]. Overall these findings therefore suggest that amygdala hyperactivity is inherited in those at familial risk for bipolar disorder and that further regional dysfunction in the insula cortex is involved in those who subsequently convert to mood disorder.

Regarding the clinical outcome of the ill individuals, previous longitudinal studies of the offspring of parents with BD have similarly found that a significant proportion develop unipolar depression [34]. Also, of offspring that develop BD, almost all experience depression or other mood disorders years before conversion [34], [35]. It is therefore likely that some of our MDD participants may in future develop BD. The time course for this shift is unknown and follow-up of these individuals will contribute to our understanding of the pathway to illness.

Finally, sub-syndromal Hamilton Depression rating scores were highest in individuals who later developed MDD. It is possible that some individuals in the group that were subsequently diagnosed as having MDD may have been prodromal at the time of the baseline scan. However, the Hamilton Depression rating scores were *also* significantly higher in the HR well group compared to controls and did not differentiate the high risk groups themselves. This suggests an overall raised level of sub-clinical symptoms in the high-risk group as a whole, rather than being specific to those who subsequently became ill. This finding is consistent with other studies showing elevated depression symptom scores in relation to elevated genetic risk [36]. It is also worth noting that the baseline HAM-D scores did not correlate with the measures of insula activation, whereas trait measures of neuroticism, cyclothymia and extraversion did. This suggests that individual differences in the levels of insula activation were not simply a consequence of depressive symptoms.

In summary, these findings demonstrate that dysfunction of the insula, a region known to be involved in mood regulation, is present at baseline in high-risk subjects who later develop a mood disorder. Activation differences distinguished those who developed MDD from controls, and from those at familial risk who did not become unwell. These findings advance our understanding of the biological processes involved in the development of mood disorders and provide a potential biomarker that could be tested for clinical utility in future studies.

## Supporting Information

Text S1 **Methods.**
(DOC)

Table S1 **Pair-wise comparisons of clinical, behavioural and temperament measures.**
(DOC)

Figure S1 **Main task-related activations and deactivations.** Depicts regions of activation for the contrast of [sentence completion versus baseline] in red, and [baseline versus sentence completion], or ‘deactivations’, in blue, demonstrating a relative decrease in activation during the task in the midline fronto-parietal regions, bilateral insula cortex and amygdala. Maps determined using within group random effects analysis of controls and high risk individuals combined. Images are overlaid onto standard brain in MNI space using Mango software package (http://ric.uthscsa.edu/mango). Map represents T-statistic images thresholded equivalent to p uncorrected = 0.001 (scaled T = 3 to 5).
(DOC)

Figure S2 **Parametric activations for group separately.** Depicts regions of activation for the parametric contrast for controls (red), bipolar high-risk well (green) and bipolar high-risk ill (blue). Images are overlaid onto standard brain in MNI space using Mango software package (http://ric.uthscsa.edu/mango). Map represents T-statistic images thresholded equivalent to p uncorrected = 0.001. (scaled T = 3 to 5).
(DOC)
