# Supplementary material for: Prediction of Depression in Individuals at High Familial Risk of Mood Disorders Using Functional Magnetic Resonance Imaging
Source: PLoS One. 2013 Mar 6;8(3):e57357. doi: 10.1371/journal.pone.0057357 (PMC3590244; doi:10.1371/journal.pone.0057357)
Supplement: Text S1 — Methods. (DOC) [file pone.0057357.s001.doc]

**Supplementary Text S1**

Scanning procedure

Imaging was carried out at the Scottish Brain Imaging Research Centre on a GE 1.5 T Signa scanner (GE Medical, Milwaukee, USA). The functional imaging protocol consisted of axial gradient-echo planar images (EPI) (TR/TE = 2000/40ms; matrix = 64x64; field of view = 24cm) acquired continually during the experimental paradigm. Twenty-seven contiguous 5 mm slices were acquired within each TR. Each EPI acquisition was run for 404 volumes. The first 4 were discarded. The T1 sequence yielded 180 contiguous 1.2 mm coronal slices (matrix = 192 x 192; fov = 24 cm; flip angle 8°).

Image processing and analysis

EPI and T1 images were reconstructed into NIFTI format (Mayo Foundation, Rochester, MN, USA) using DICOM convert functions available in SPM5 (Statistical Parametric Mapping; http://www.fil.ion.ucl.ac.uk/spm/) running in Matlab (The MathWorks, Natick, MA, USA). EPI images were realigned to the mean functional image to correct for movement. The structural (source) and functional (reference) image were then co-registered and the anatomical image was then segmented. Spatial normalisation parameters generated from the previous step were used to normalise the realigned functional EPI data. Finally the realigned normalised images were smoothed with an 8x8x8mm full width half maximum Gaussian filter.

First level analysis

Statistical analysis was performed using the general linear model approach as implemented in SPM5. At the individual subject level the data was modelled with 4 conditions corresponding to the four difficulty levels each modelled by a boxcar convolved with a synthetic haemodynamic response function. Estimates of the subject’s movement were entered as ‘covariates of no interest’. The design matrix included a high pass filter (128s) and serial correlations were accounted for by using the autoregressive (AR(1)) model. Contrasts were constructed to examine all four sentence completion conditions versus baseline, and areas of increasing activation with increasing task difficulty (the parametric contrast).

Example sentences where 1-4 represents increasing difficulty:

(1) High constraint: “He mailed the letter without a ........”

(2) Medium high constraint: “The train was still on ........”

(3) Medium low constraint: “Not even the cast liked the ........”

(4) Low constraint: “Rushing out he forgot to take his ........”
