# Supplementary material for: Prediction of Depression in Individuals at High Familial Risk of Mood Disorders Using Functional Magnetic Resonance Imaging
Source: PLoS One. 2013 Mar 6;8(3):e57357. doi: 10.1371/journal.pone.0057357 (PMC3590244; doi:10.1371/journal.pone.0057357)
Supplement: Table S1 — Pair-wise comparisons of clinical, behavioural and temperament measures. (DOC) [file pone.0057357.s002.doc]

**Supplementary Table S1** *Pair-wise comparisons of clinical, behavioural and temperament measures*

|  | **Controls versus Bipolar high-risk well** | | **Controls versus Bipolar high-risk subsequently ill** | | **Bipolar high-risk well versus high-risk subsequently ill** | |
| --- | --- | --- | --- | --- | --- | --- |
|  | P value | χ^2^ | P value | χ^2^ | P value | χ^2^ |
| HAM-D | 0.040 | 4.21 | 0.004 | 11.15 | 0.052 | 3.79 |
| TEMPS-A: cyclothymia | 0.382 | 0.76 | 0.001 | 13.91 | <0.001 | 12.14 |
| TEMPS-A: depressive | 0.498 | 0.46 | 0.009 | 9.45 | 0.002 | 9.30 |
| TEMPS-A: irritability | 0.977 | 0.01 | 0.012 | 8.88 | 0.004 | 8.50 |
| NEO – FFI: neuroticism | 0.674 | 0.18 | 0.001 | 14.11 | 0.001 | 11.75 |
| NEO – FFI: extraversion | 0.068 | 3.33 | 0.10 | 9.21 | 0.038 | 4.28 |
| NEO – FFI: conscientiousness | 0.415 | 0.67 | 0.046 | 6.16 | 0.039 | 4.25 |
