# Supplementary material for: Prediction of Depression in Individuals at High Familial Risk of Mood Disorders Using Functional Magnetic Resonance Imaging
Source: PLoS One. 2013 Mar 6;8(3):e57357. doi: 10.1371/journal.pone.0057357 (PMC3590244; doi:10.1371/journal.pone.0057357)
Supplement: Figure S1 — Main task-related activations and deactivations. Depicts regions of activation for the contrast of [sentence completion versus baseline] in red, and [baseline versus sentence completion], or ‘deactivations’, in blue, demonstrating a relative decrease in activation during the task in the midline fronto-parietal regions, bilateral insula cortex and amygdala. Maps determined using within group random effects analysis of controls and high risk individuals combined. Images are overlaid onto standard brain in MNI space using Mango software package (http://ric.uthscsa.edu/mango). Map represents T-statistic images thresholded equivalent to p uncorrected = 0.001 (scaled T = 3 to 5). (DOC) [file pone.0057357.s003.doc]

**Supplementary Figure S1** *Main task-related activations and deactivations*


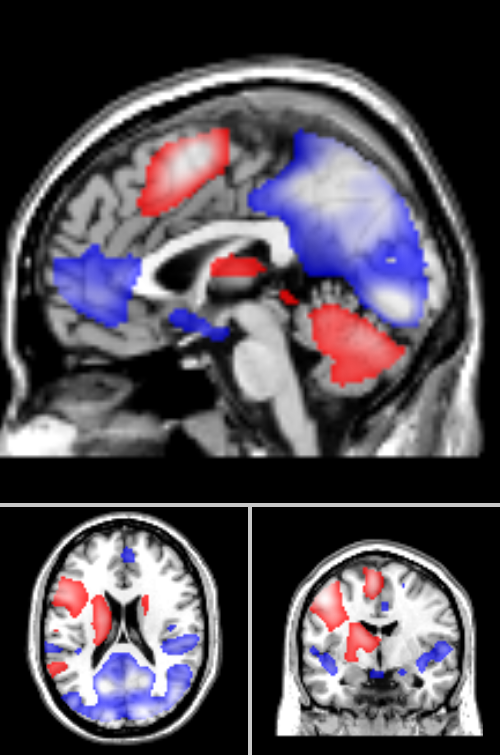


Depicts regions of activation for the contrast of [sentence completion versus baseline] in red, and [baseline versus sentence completion], or ‘deactivations’, in blue, demonstrating a relative decrease in activation during the task in the midline fronto-parietal regions, bilateral insula cortex and amygdala. Maps determined using within group random effects analysis of controls and high risk individuals combined. Images are overlaid onto standard brain in MNI space using Mango software package (<http://ric.uthscsa.edu/mango>). Map represents T-statistic images thresholded equivalent to p uncorrected=0.001 (scaled T=3 to 5).
