# Supplementary material for: Prediction of Depression in Individuals at High Familial Risk of Mood Disorders Using Functional Magnetic Resonance Imaging
Source: PLoS One. 2013 Mar 6;8(3):e57357. doi: 10.1371/journal.pone.0057357 (PMC3590244; doi:10.1371/journal.pone.0057357)
Supplement: Figure S2 — Parametric activations for group separately. Depicts regions of activation for the parametric contrast for controls (red), bipolar high-risk well (green) and bipolar high-risk ill (blue). Images are overlaid onto standard brain in MNI space using Mango software package (http://ric.uthscsa.edu/mango). Map represents T-statistic images thresholded equivalent to p uncorrected = 0.001. (scaled T = 3 to 5). (DOC) [file pone.0057357.s004.doc]

**Supplementary Figure S2** *Parametric activations for group separately*


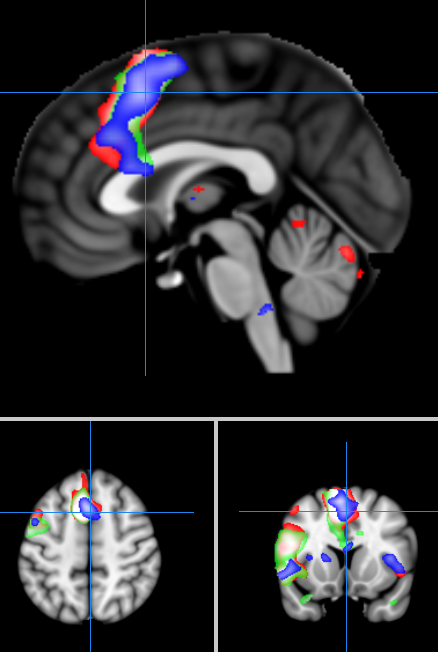


Depicts regions of activation for the parametric contrast for controls (red), bipolar high-risk well (green) and bipolar high-risk ill (blue). Images are overlaid onto standard brain in MNI space using Mango software package (<http://ric.uthscsa.edu/mango>). Map represents T-statistic images thresholded equivalent to p uncorrected=0.001. (scaled T=3 to 5).
